# RaVÆn: unsupervised change detection of extreme events using ML on-board satellites

**DOI:** 10.1038/s41598-022-19437-5

**Published:** 2022-10-08

**Authors:** Vít Růžička, Anna Vaughan, Daniele De Martini, James Fulton, Valentina Salvatelli, Chris Bridges, Gonzalo Mateo-Garcia, Valentina Zantedeschi

**Affiliations:** 1 University of Oxford, Oxford, UK; 2 University of Cambridge, Cambridge, UK; 3 University of Edinburgh, Edinburgh, UK; 4 Microsoft Research, Cambridge, UK; 5 University of Surrey, Guildford, UK; 6 University of Valencia, Valencia, Spain; 7 ServiceNow Research, Canada, University College London, London, UK; 8 Frontier Development Lab, Oxford, UK

**Keywords:** Environmental sciences, Natural hazards, Computer science, Scientific data, Software

## Abstract

Applications such as disaster management enormously benefit from rapid availability of satellite observations. Traditionally, data analysis is performed on the ground after being transferred—downlinked—to a ground station. Constraints on the downlink capabilities, both in terms of data volume and timing, therefore heavily affect the response delay of any downstream application. In this paper, we introduce **RaVÆn**, a lightweight, unsupervised approach for change detection in satellite data based on Variational Auto-Encoders (VAEs), with the specific purpose of on-board deployment. **RaVÆn** pre-processes the sampled data directly on the satellite and flags changed areas to prioritise for downlink, shortening the response time. We verified the efficacy of our system on a dataset—which we release alongside this publication—composed of time series containing a catastrophic event, demonstrating that **RaVÆn** outperforms pixel-wise baselines. Finally, we tested our approach on resource-limited hardware for assessing computational and memory limitations, simulating deployment on real hardware.

## Introduction

Satellite observations of the Earth’s surface provide vital data for diverse environmental applications, including disaster management^1,2^, landcover change detection^3^, and ecological^4^, urban^5^ and agricultural^6^ monitoring. Currently, Earth observation (EO) satellites collect and downlink raw or low-compression-rate images for further processing on the ground^7^. Limitations in downlink capacity and speed result in delayed data availability and inefficient use of ground stations. This adversely impacts time-sensitive applications such as disaster management where data is required at low latency to inform time-critical decision making. This problem is set to worsen as the sensing resolution and number of EO satellites in orbit increase^8^, together with further restrictions on the radio-frequency spectrum and licensing availability.

One solution is to identify on-board the most useful data for a particular scenario and prioritise this for rapid downlink. Although on-board processing of payload data using machine learning has long been recognised as a potential method to improve efficiency^9,10^, recent advances in hardware and machine learning that make deployment feasible have lead to a resurgence of interest in this area^7^. In recent years supervised classifiers have successfully been tested in orbit to segment clouds^11,12^ and floods^13^, with proposed applications such as storm identification^14^. Still, supervised classifiers have the significant drawback in that only events of a particular type determined at training time will be identified. The model is therefore unable to generalise to new event types, imager specifications, sensor degradation, scene lighting or local features.

In this work, we present **RaVÆn** , a new fully-unsupervised novelty-detection model that avoids the limitations inherent in supervised classifiers and is suitable for deployment on remote sensing platforms. We use a *Variational Auto-Encoder* (VAE)^15^ to generate a latent representation of incoming sensor data over a particular region. A novelty score is assigned to this data using the distance in the latent space between representations from consecutive passes. This technique offers a substantial advantage over existing supervised methods as any change between passes can be detected on-board, regardless of the availability of training data for specific event types. Furthermore, even in situations of compound events, this general approach supports the detection of all types of changes present in the region.

We evaluate the performance of this model in detecting changes in land-surface observations from the Sentinel-2 Multispectral Instrument^16^ on a dataset of time series of images of natural disasters. Four event types where rapid response by emergency agencies is essential are included: floods, landslides, wildfires and hurricanes. **RaVÆn**  is demonstrated to assign higher novelty scores to regions of known change, and outperforms classical image differencing computer-vision baselines requiring 60$\times$ less onboard storage. We further demonstrate via experiments on constrained hardware that emulates on-board processors that this model is suitable for deployment on a remote sensing platform.

The rest of the paper is organized as follows: in “Background” we frame our proposal in the context of machine learning and satellite onboard deployment, in “Data” we present the datasets that we used for training the model and the test dataset collected for this work which is made public with this paper, in “Methodology” the proposed methodology for change detection and baseline models are presented, “Experimental setup” discusses the metrics and hardware that we used for the experiments; finally, “Results” shows the results of the models and baselines on the annotated dataset and benchmarks the proposed model on constrained hardware and “Conclusion” summarises the conclusions of the paper.

## Background

### Anomaly detection

The use of VAEs has been explored for unsupervised anomaly detection in^17^, where the model reconstruction error is used as anomaly score. Our approach differs in that, instead of basing our predictions on the reconstruction error of a single input, which has been shown in^18^ to be an unreliable indicator in the unsupervised context, we consider a sequence of input images from the same location and score them based on their distance in the VAE’s latent space. We could also represent a sequence of images (or extracted tiles) as a single data point, where we would later use methods such as for example Reed-Xiaoli^19^, or Mixture of Gaussians to detect anomalous and background sequences. Our problem is however better framed as change detection.

### Change detection

The need for annotations of supervised change detection techniques, such as siamese networks in^20^, can be reduced using active learning approaches as demonstrated in^21^, but then it still lacks in terms of generality. The main challenge of unsupervised change detection is being able to distinguish changes of interest from spurious change due to noise. Many existing approaches^22–24^ achieve this by combining dimensionality reduction techniques, such as Principal Component Analysis^25,26^, and clustering, such as *k*-means, to detect only relevant change between images of consecutive passes. Approaches based on neural networks (see^27^ for a review) rely instead on supervised auxiliary tasks, such as semantic segmentation, to extract informative features that are then used to detect change in a time series. Our method leverages neural networks without requiring supervision at any stage. Our chosen method is closest to the work of^28^, however the focus of on-board deployment is novel.

### ML deployment on satellites

Deploying machine learning models onboard remote sensing platforms has been identified as a potential solution to optimize downlinking communication and onboard storage^13,29,30^. Most of current public and commercial missions such as e.g. ESA PRISMA^31^ or Maxar’s WorldView-3 acquire images on demand when they are tasked from the ground (specifically when they flight over an specific area of interest). Even global missions, such as Sentinel-1 or Sentinel-2 acquire images only over certain pre-configured conditions (more frequently over Europe and over land locations respectively). Our proposal demonstrates that with relatively simple ML models we can deploy autonomous algorithms to decide onboard which tiles within an acquisition shall be downlinked based on the retrieved observation. With this system we aim to showcase a change of paradigm in Remote Sensing operations: from a regime where sensors acquire and downlink data based on *ad-hoc* manual configurations to autonomous acquisitions where the sensing platform continuously evaluate if retrieved data has value to prioritise its download or discard it.

## Data

**Figure 1:**
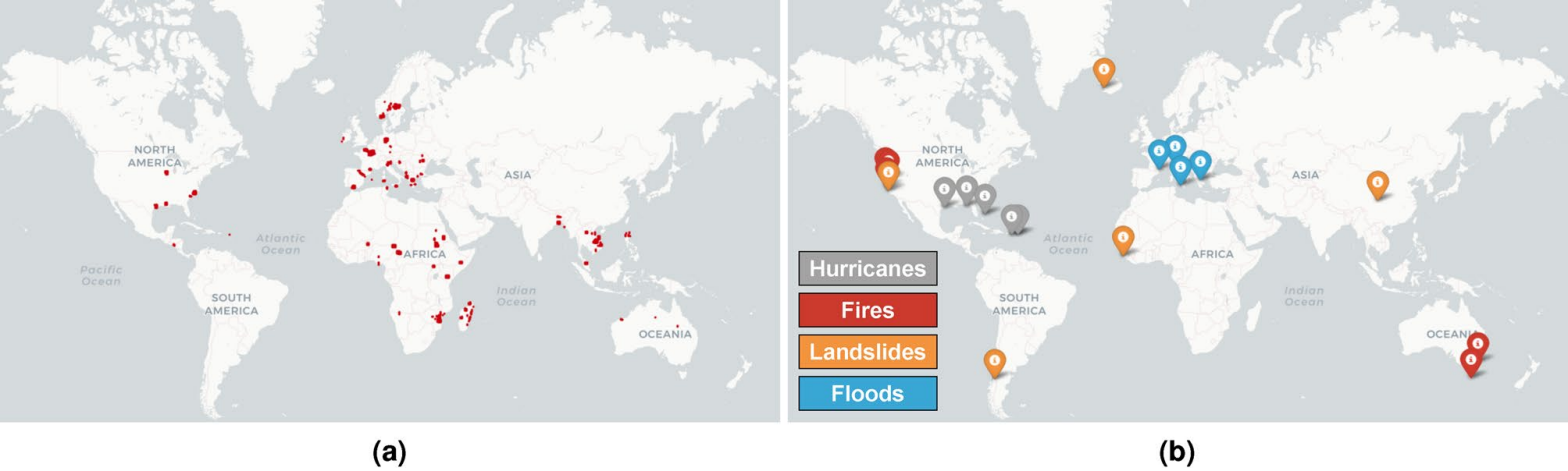
Locations used for training (**a**) and validation (**b**) images.

As part of this study, we compile and release a new dataset to evaluate the proposed unsupervised change detection models. Images are taken from the Sentinel-2 multi-spectral imager (MSI) instrument^16^ (using the L1C processing level of the data) from which we use the ten highest resolution channels with all channels interpolated to the highest resolution of 10m. Training data are taken from the *WorldFloods* dataset^13^ (Fig. 1a), with a total of 233 scenes and a time series of five images per scene.

**Figure 2:**
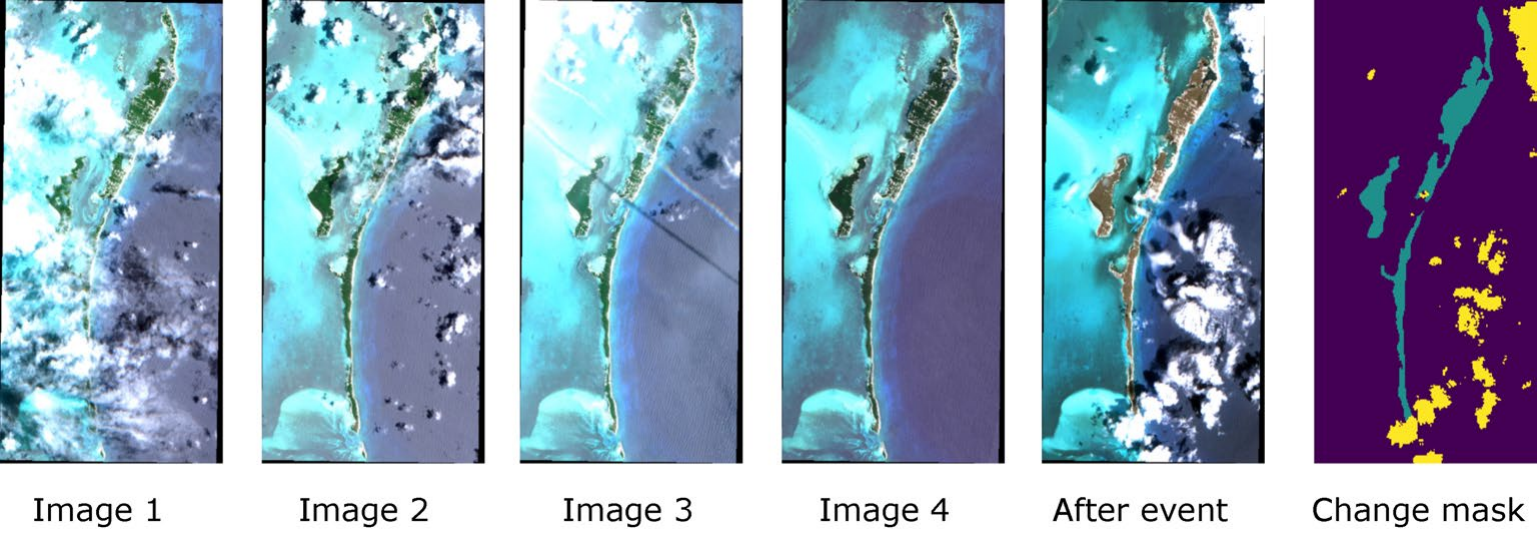
Example of validation sample—in this case, a hurricane event—and its corresponding ground-truth mask (which contains labels of change and clouds).

### The RaVÆn dataset

The validation set consists of 19 scenes captured from Sentinel-2, containing one of four classes of disasters: hurricanes, fire burn scars, landslides and floods (Fig. 1b). We identified events in each of these classes through an extensive search of Sentinel-2 records aided by the Copernicus EMS system^32^. Each event in the validation set consists of a time series of five images where the first four images are taken before the disaster occurred while the fifth image is taken afterwards. To mitigate the effects of cloud cover, we discarded validation images with greater than 20% cloud cover. Events are only included where all images are within 180 days before and 90 days after the event. For each event a change mask was hand annotated to mark differences between the final two images in the time series, as in Fig. 2. Cloud cover generated using s2cloudless^33^ and invalid pixels were also annotated in the change masks. We emphasize that these labels are used for evaluation only.

We describe the statistics of the manually annotated validation dataset in Table 1. While each type of event is represented by a similar amount of locations, the affected area varies significantly depending on the disaster type. Namely the area of burn scars in the *Fire* dataset has both the largest area of effect and the largest proportion of changed pixels to all non-cloudy pixels (reported as positive ratio).

**Table 1:** The RaVÆn dataset statistics.

	Number of locations	Cumulative area (km$^2$)	Positive rate
Landslides	5	108	10.48
Floods	4	1301	6.74
Hurricanes	5	1622	24.31
Fires	5	3485	53.79

Each location is captured in 4 time-steps before the event and once after the event. Positive rate denotes the ratio of changed to non-cloudy pixels in the last pair of images (the only frames that are annotated).

## Methodology

### Preprocessing

**Figure 3:**
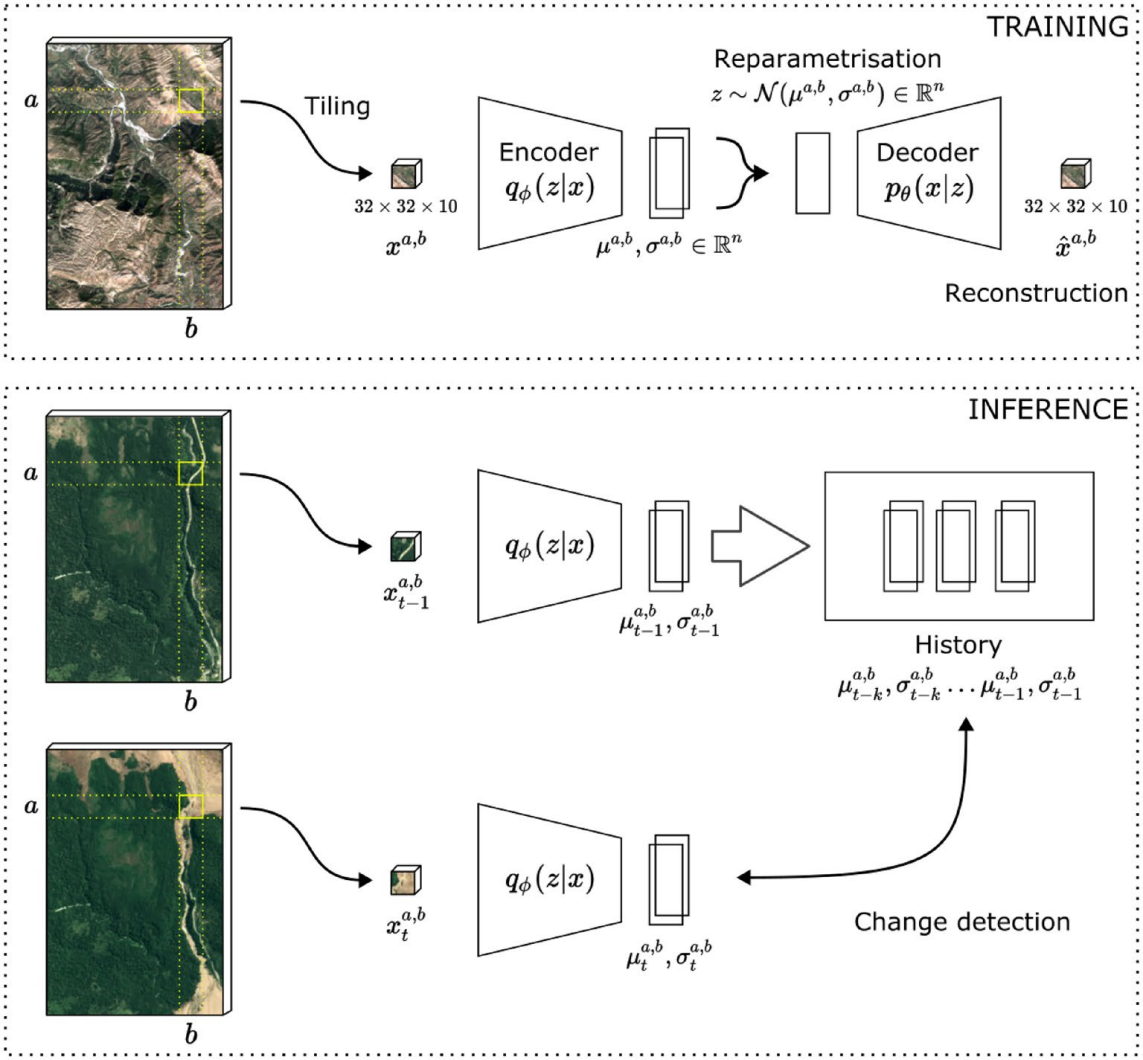
Diagram of the proposed system. Tiles $x^{a,b}$ of dimension $32 \times 32 \times 10$ from the original Sentinel-2 multiband L1C data from the training dataset are fed to a VAE model. Here, *a* and *b* correspond to the location of the tile. The VAE is trained in an unsupervised fashion as its encoder learns to compress the tile in an Gaussian embedding representation $\mu ^{a,b}$ and $\sigma ^{a,b}$ and the decoder to reconstruct them from there. At inference, only the trained encoder is needed as we compress evalutation dataset tiles $x^{a,b}_t$ into their embeddings $\mu ^{a,b}_t$ and $\sigma ^{a,b}_t$ which can be compared against an history of *k* embeddings extracted at the same location to assess whether the tile has changed significantly and prioritise for downlink.

Tiles $x^{a,b}$ of $32 \times 32$ pixels—and therefore 320m $\times$ 320m area—are extracted from the Sentinel-2 scenes as shown in Fig. 3 and used as inputs to the considered model. Here, *a* and *b* represent the location of the tile in the scene.

The tiles are further normalized by applying a log transform and scaling to constrain them to the $[-1,+1]$ interval using the following transformation for each band:

1 $$\begin{aligned} x'= & \,log(x)\nonumber \\ x''= & \,2 * \frac{x' - min(x')}{max(x') - min(x')} - 1 \end{aligned}$$

Values for min and max were selected manually based on visual analysis of the training data distribution and fixed for all experiments. Note that multiplication and subtraction are pixel-wise. These preprocessing steps are consistent with other papers^34^. On-board satellites, imager sampling and memory interfacing regimes vary and this work omits to match our architecture to any one sampling method.

### Model

We employ a Variational Auto-Encoder (VAE) model, as shown in Fig. 3, to learn a low-dimensionality embedding space for tiles $x^{a,b}$ then exploited for change detection. A generic Auto-Encoder (AE) model consists of two networks—called *encoder* and *decoder*—usually composed out of convolutional or fully-connected layers of neurons. The encoder network $q_\phi$ learns to project the data from the original domain into a generally lower dimensional “bottleneck” representation—called *latent space*—while the decoder network $p_\theta$ learns to reconstruct the original data from this latent vector representation. The whole model learns end-to-end and doesn’t require any specific labels, as the task is to reconstruct the original data while obtaining a representative latent space. Given the fact that this latent space is of smaller dimensionality than that of the original data, it can be understood as a compressed space, with only the distinguishing features present in the representation. This learned latent space can be used for further downstream tasks, such as for training with labelled data (in the case of our data, this would for example be in classification of tiles to cloudy or non-cloudy tiles), or for change detection via comparison of the embedded latents, as is further described in this paper. VAEs impose further restrictions on the distribution of the learned latent variables as is detailed in^15^. In particular, the VAE imposes a diagonal Gaussian distribution for the embeddings that is parameterized by the mean and standard deviation of each dimension, ${\mathcal {N}}^{a,b} = {\mathcal {N}} (\mu ^{a,b}, \sigma ^{a,b})$. The latent representation $\mu ^{a,b}, \sigma ^{a,b} \in {\mathbb {R}}^n$, where *n* is called *latent size*.

### Change detection novelty score

At inference, we can drop the decoder $p_\theta$ and use only the trained encoder network $q_\phi$ as a feature extractor to encode individual tiles in their compressed representation, with the advantage of improved robustness to noise and to slight misalignment between tiles^35^ and reduced computational and memory requirements of storing images from previous passes, which is a critical in a constrained environment.

More formally, given a new tile $x^{a,b}_t$ at time t, we seek to understand if a relevant change has happen from the *k*-long history of samples at the same location $\{x^{a,b}_{t-k},\dots x^{a,b}_{t-1}\}$. To achieve this, we define a novelty score function $S({\mathbb {R}}^{32\times 32\times 10}) \rightarrow {\mathbb {R}}$ as:

2 $$\begin{aligned} S(x_t^{a,b}) = \min _{i = 1\ldots k} d(x_{t-i}^{a,b},\, x_t^{a,b}) \end{aligned}$$

where *d*$({\mathbb {R}}^{32\times 32\times 10}, {\mathbb {R}}^{32\times 32\times 10}) \rightarrow {\mathbb {R}}$ is an arbitrary difference function between two tiles. We propose to employ the latent representation of the tiles within *d* and we test three different distance functions in “Results”, the Euclidean and cosine distance between the means $\mu ^{a,b}_t$ and $\mu ^{a,b}_{t-i}$, and the KL divergence between the Gaussian latents ${\mathcal {N}}^{a,b}_t$ and ${\mathcal {N}}^{a,b}_{t-i}$. In Eq. (2) we use the minimum as a function to aggregate the individual distances, with the assumption that it is the last sample of the time-series of tiles, that contains the relevant change. This helps us ignore small fluctuations in the previous tiles.

### Compression

In this analysis, we fix the latent size to $n=128$ as initial experiments indicated that larger latent sizes did not yield improved results and that lower values decreased the model performance. This gives us significant compression capabilities when deployed, as, instead of saving the original images or the extracted tiles ($32 \times 32$ image of 10 bands with 12 bits as per the Sentinel-2 radiometric resolution specification), the application simply stores their computed latent representations (in some cases only the encoded mean vector 128 with 16 bit float precision). This approach provides users with 60$\times$ reduction of necessary storage space with the caveat that further compression of the latent vectors is possible^36^. When comparing this solution against existing satellite practices, the a typical EO satellite predominantly use lossless compression such as CCSDS-123^37^ or and JPEG-LS^38^. These achieve compression ratios of approximately 6.5:1 and 2.5:1 respectively. Our solution is lossy and the original raw pixel values cannot be fully reconstructed. Unlike existing lossless systems, our approach ensures accurate information can be utilised quickly and effectively in complementing real-time decision making systems.

### Preprint

A shorter version of this paper was previously presented at the Artificial Intelligence for Humanitarian Assistance and Disaster Response Workshop (HADR) at NeurIPS 2021 (virtual)^39^. The pre-print was peer-reviewed for inclusion in the workshop, which is not archival and does not form part of the NeurIPS conference proceedings. This paper has been updated with follow-up ideas and significantly restructured.

## Experimental Setup

### Model architecture design

The encoder of our VAE was composed of a series of downsampling blocks. Each downsampling block first had a 2D convolutional layer with kernel size 3, stride size 2, and zero padding of 1, such that the dimensions are halved in the spatial domain. Following this layer, the block also had a sequence of extra 2D convolutional layers (the number *extra depth* referred to in Table 2). Skip connections were used so that the *extra depth* convolutional layers formed a residual block. The network could then easily learn to skip these non-downsampling layers. In the residual block, the number of hidden channels and image size were conserved. Each convolution layer used leaky ReLU activations and batch normalisation. Following a given number of downsampling blocks, the result was flattened and further reduced in dimension using a fully connected layer which outputs the mean and log variance. The decoder was essentially the encoder in reverse. The upsampling method used was nearest neighbour upsampling followed by a single convolution. This method was preferred over transpose convolution to avoid checkerboard artefacts^40,41^. Last layer of the decoder network uses a linear activation function to allow for reconstruction in the original data range. Finally, for training we use the Adam optimizer and learning rate of 0.001.

### Efficiency considerations

To optimize the size of the model and maximize efficiency on constrained devices we conducted a parametric search over both the number of layers and number of units per layer in both the encoder network *E* and the decoder network *D*. More precisely we tested three different model architecture configurations (*small*, *medium* and *large*) detailed in Table 2. The main model presented in this paper is denoted as *large* on Table 2, it used 3 downsampling blocks with 32, 64, 128 channels on each successively smaller scale. A final fully connected layer projects the input to a latent dimension of 128. After each downscale convolution there was a residual block of 2 (extra depth) additional convolutional layers.

### Hardware deployment

We use different environments for training the VAE and for inference. For development (training and validation), we use a n1-standard-16 instance on Google Cloud Platform with two NVIDIA Tesla V100 GPUs. In addition we measure the performance of the models on the Xilinx Pynq FPGA board with limited compute power, 650 MHz ARM Cortex-A9 CPU and 512 MB RAM which emulates the resources available on a typical small satellite (motivated by^42^).

**Table 2:** Differences in the architecture for different proposed model sizes.

	Total params. (millions)	Encoder params. (millions)	Extra depth	Hidden channels	Latent size
Small model	0.443	0.285	0	16, 32, 64	128
Medium model	0.979	0.617	0	32, 64, 128	128
Large model	1.463	1.007	2	32, 64, 128	128

Note that during inference, we only need the encoder network of the VAE model. We also only need to process the newly acquired image to obtain their latent representation, while the latent vectors of the previous image can be loaded.

### Baselines

To compare the performance of this approach to simpler on-board processing methods that does not make use of machine learning, we compare our method to a baseline which compares tiles directly in the input space using the Euclidean or the cosine distance and after applying the same data pre-processing as for the VAE.

## Results

**Figure 4:**
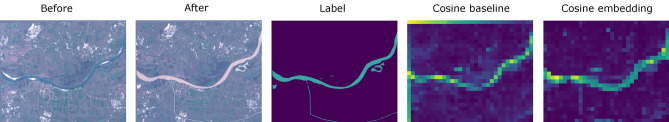
Comparison of the change detected using the baseline and the *large* VAE method on an example of a flooding river. Two images immediately before and immediately after a change are shown, along with the human labels of change and the calculated change scores. Both methods used a history of $k=3$ frames.

**Figure 5:**
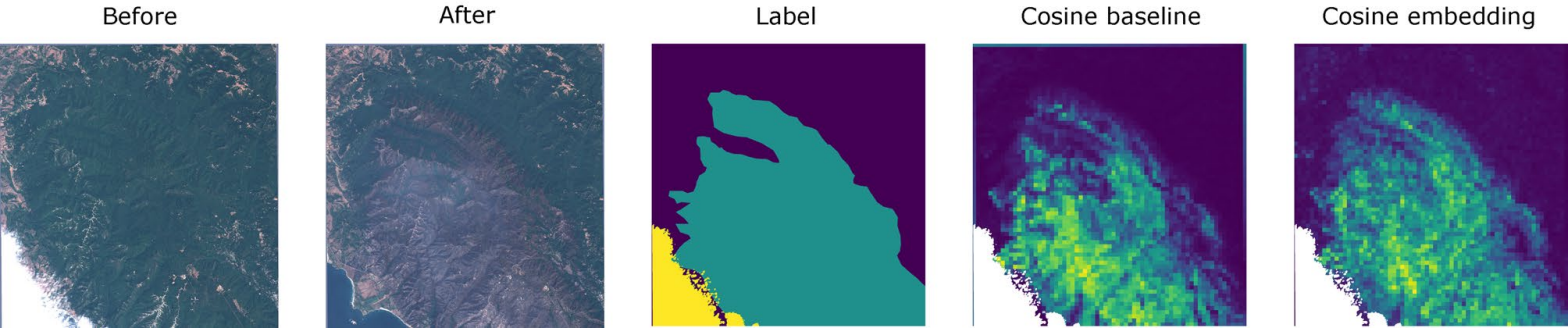
Additional comparison of the change detected using the baseline and the *large* VAE method on an example of a fire disaster. Both methods used a history of $k=3$ frames. The cosine baseline prediction seems to more closely copy the details present in the image, making it susceptible to small, noisy variations between the two images.

Figure 4 shows a qualitative comparison between the VAE model developed in this study and the image differencing baseline. The *before* image shows a river that floods and therefore changes colour in the *after* image. The labels and the change scores from our VAE and baseline methods are shown alongside. In this case, the scores were calculated using a history of $k=3$ frames, although only the most recent *before* frame is shown for brevity. In this example, our method—the cosine embedding—produces a change map that is crisper than the cosine baseline; notably, the small flooded canal can be seen in the cosine embedding image but not in the baseline. In a similar fashion, Fig. 5 shows a qualitative comparison in the case of a burnt-area detection.

The change-score maps, like those in Figs. 4 and 5, were produced for every image in the evaluation set. We use these maps and our labels to calculate the area under the precision-recall curve (AUPRC). We produce the curve tile-wise, so that each individual tile across each image is treated as a positive or negative example of change, rather than treating the full image as one example. This means our quality metric is sensitive to the fact that our evaluation images are not equal; they have different number of tiles and different ratios of positive pixels (as reported in Table 1). We also ignore tiles that have clouds in the *after* image or in the most recent image before the event. We produce a precision-recall curve for each of the four different event types in our evaluation set and calculate separately the AUPRC.

We further note, that the used AUPRC metric does not require a specific threshold to be selected. In practice, the threshold would be selected based on operational constraints. For example, if only a certain number of tiles were able to be downlinked from a remote sensing platform, then the tiles with the largest change value would be selected for transmission.

Table 3 reports the results of our change detection experiments for all disaster types. We found that cosine distance, applied on the input space or on the embeddings, generally provides the best scores. This is in accordance with other research, which uses the cosine distance for comparisons in the latent space^43^ or when training contrastive learning methods^44^. For the metrics based in the embedding space, there was some variation between event types. Surprisingly KL-divergence is the lowest-performing metric, and is beaten by both cosine and Euclidean embedding scores in all events, even though these methods do not use the variance values calculated by the VAE. Metrics based on the VAE embedding outperforms the baseline on floods, hurricanes and fires, and reaches similar performance on landslides.

Table 4 shows the effects of including a longer frame history. When three previous images are provided instead of just one, both the embedding and baseline perform better except for the case of landslide dataset where the cosine baseline with memory 1 beats memory 3 with a small margin. The table also shows that our method of detecting significant change based on the embedding space outperforms the baselines in every dataset when $k=3$ by a large margin.

**Table 3:** AUPRC for baseline and VAE methods with time window $k=1$ (averaged over 5 runs).

Detection method	Dataset			
	Landslides	Floods	Hurricanes	Fires
Cosine baseline	**0.629**	0.378	0.513	0.818
Euclidean baseline	0.267	0.326	0.351	0.770
Cosine embedding	0.599 ± 0.012	**0.448** ± 0.011	**0.676** ± 0.014	**0.833** ± 0.008
Euclidean embedding	0.266 ± 0.004	**0.450** ± 0.007	0.478 ± 0.019	0.800 ± 0.011
KL-Divergence	0.258 ± 0.022	0.247 ± 0.018	0.301 ± 0.035	0.731 ± 0.016

**Table 4:** AUPRC for the best performing metrics from Table 3 with and without an extended history *k* (averaged over 5 runs).

**Detection method**	**k**	**Dataset**			
		Landslides	Floods	Hurricanes	Fires
Cosine baseline	1	0.629	0.378	0.513	0.818
	3	0.622	0.378	0.570	0.865
Cosine embedding	1	0.599 ± 0.012	**0.448** ± 0.011	0.676 ± 0.014	0.833 ± 0.008
	3	**0.759** ± 0.024	**0.443** ± 0.009	**0.726** ± 0.011	**0.913** ± 0.008

### Model timings and memory footprint

The purpose of the proposed change detection method is to run onboard a satellite, to be used for filtering or prioritising the image tiles to be downlinked. Therefore, models need to be designed to keep up with the upcoming stream of data on constrained, low-power hardware similar to the available on real remote sensing satellites. Here we report the accuracy and inference time of the different models architectures (see Table 2).

**Table 5:** AUPRC and timings for different sizes of model (averaged over 5 runs).

	Dataset				Runtime (seconds)
	Landslides	Floods	Hurricanes	Fires	
Small model	**0.748** ± 0.014	**0.445** ± 0.014	**0.748** ± 0.002	0.907 ± 0.002	2.06
Medium model	**0.758** ± 0.007	0.428 ± 0.004	**0.738** ± 0.018	**0.912** ± 0.003	4.86
Large model	**0.759** ± 0.024	**0.443** ± 0.009	0.726 ± 0.011	**0.913** ± 0.001	13.98

The AUPRC results are for the cosine similarity of the embedding with a history of 3 frames. Runtime is measured on-board of the Xilinx Pynq board.

Table 5 shows the accuracy of a few variations of model size and the time it took to process a 574 $\times$ 509 px image (approx. 5 km $\times$ 5 km at Sentinel-2 10m resolution) whilst running on the CPU of a Xilinx PYNQ. We see the the results of all tested models are comparable and that it is reasonable to aim for the smallest model, which takes only 2.06 s to process the patch. Running onboard the PYNQ means that there is considerable potential to speed up this runtime by a large factor by deploying directly on the FPGA module rather than using the board’s CPU.

Additionally, we report that executing our code on the device left at least 67% of the total RAM available for other processes (we note that this includes any other background processes that would run alongside our code on clean Pynq environment).

**Table 6:** AUPRC and for models with different latent sizes (averaged over 5 runs).

Latent size	Dataset				Total params. (millions)
	Landslides	Floods	Hurricanes	Fires	
128	**0.759** ± 0.024	**0.443** ± 0.009	**0.726** ± 0.011	**0.913** ± 0.001	1.463
96	0.723 ± 0.005	0.419 ± 0.010	0.687 ± 0.034	0.905 ± 0.004	1.266
64	0.699 ± 0.015	0.392 ± 0.017	**0.726** ± 0.022	0.903 ± 0.006	1.069

The AUPRC results are for the cosine similarity of the embedding with a history of 3 frames. Models use parameters for the default large model, but use variable latent sizes. We also show the total number of parameters of each model.

Table 6 shows the experiments with changing the latent size of the default model (denoted as the “Large model” in Table 2) while not altering any other architectural hyper-parameters. We see, that with decreased latent size we encounter drop in performance for most of datasets. We use these results to fix the latent size to $n = 128$ in all other experiments.

### Latent space visualisation

**Figure 6:**
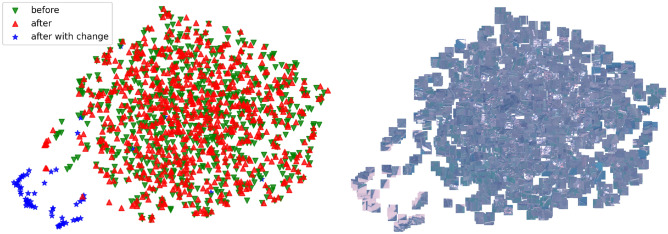
UMAP visualisation of encoded tiles from flooded scene presented on Fig. 4. Tiles from the image before the event are marked as green, while the tiles from after the event are shown in red. Tiles corresponding to the flooded tiles, marked with blue, can be seen clustered together in contrast to the rest of the data from this scene.

To demonstrate the quality of learned embedding space, we show it’s graphical representations using the UMAP^45^ method in Fig. 6. We include both tiles from the image before and from the image after the event. We show that tiles of a certain type cluster together (for example the *“flooded water”* tiles). We consider this as a possible motivation for follow-up work—further using the latent representations of each tile for downstream tasks. This could be done either by unsupervised clustering, or with weak annotations of pairs of tiles corresponding to select the desired changes (whitelisting) or the changes to be ignored (blacklisting).

## Conclusion

In conclusion, we introduce a new method **RaVÆn** for unsupervised change detection in remote sensing data using a VAE. Our method is evaluated on a new dataset of remote sensing images of disasters which we release for public use with our work. The proposed model outperforms a classical computer vision baseline in all of the tested disaster classes by on average 18% (by 27% in hurricane and by 6% in fire scenarios) in the AUPRC metric when considering three past frames. This demonstrates that **RaVÆn** is a robust change detection method and suitable for application in improving data acquisition for disaster response. We also confirm that having access to longer temporal series of data can be beneficial when dealing with real-world noisy data (as also shown in^46^)—in particular having access to past three frames instead of just one previous image improves the AUPRC metric on average by 10.6% when using our proposed technique. Finally, we show that while maintaining the model’s performance ($\pm 3\%$) the model size and it’s runtime can be greatly reduced (by 85%) on the Xilinx PYNQ board, which is crucial to demonstrate the possibilities for real world deployment.

Future work could consist of using the obtained latent representations for downstream tasks such as unsupervised clustering or weakly supervised classification of types of changes (to detect or to ignore) and efficient message passing in future constellations of small satellites. Our findings also reveal several exciting research directions, such as improvements on the used metrics for measuring change between encoded latent representations. We would like to explore other unsupervised methods for learning feature extractors, such as general contrastive learning approaches like SimCLR^44^ or methods specific to Remote Sensing data such as SeCo^47^. Methods which allow for better compression of the learned latent vectors^36^ would also be beneficial for real-world application. Finally, we would also like to explore situations with access to longer temporal series of data, where it would be possible to model cyclic changes which are part of the system’s behaviour and to separate these from other detections.

## Data Availability

We are releasing the full annotated evaluation **RaVÆn** dataset, the code and the pre-trained models alongside this paper at https://github.com/spaceml-org/RaVAEn.
